# Factors Predicting Community-dwelling Older Adults’ COVID-19 Experiences in Central Texas

**DOI:** 10.1093/geroni/igab046.3206

**Published:** 2021-12-17

**Authors:** Atami Sagna de Main, Bo Xie, Kristina Shiroma, Nathan Davis

**Affiliations:** 1 The University of Texas at Austin, Austin, Texas, United States; 2 The University of Texas at Austin, The University of Texas at Austin, Texas, United States

## Abstract

Older adults, disproportionally affected by the COVID-19 pandemic, face health, social and structural vulnerabilities. Their experiences require systematic examination. Our study aimed to examine factors predicting community-dwelling older adults’ experiences during COVID-19. We collected data via the telephone between June-August 2020 from a convenience sample of older adults in Central Texas (N= 200; age range=65-92 years; Mean=73.6, SD=6.33). We conducted multinomial logistic regression analyses to model relationships between self-reported COVID-19 experiences (positive, mixed, negative) and age, gender, race, income, education, frequency of communication with family and friends, feelings of loneliness and amount of COVID-19 information obtained. Factorial analysis revealed no statistically significant interaction effect. Multinomial logistic regression analysis revealed statistically significant main effects of annual household income, feelings of loneliness and amount of COVID-19 information obtained on predicting COVID-19 experiences. Age, gender, race, education, and frequency of communication with family and friends were not significant predictors. The odds of having a positive COVID-19 experience rather than negative experiences increased by 6.94 for an annual household of $60,000- $99,999, and by 6.02 for not feeling lonely. The odds of having a positive experience during COVID-19 rather than mixed increased by 9.90 for an annual household income of $100,000 or more. Participants who reported having “too much information” about COVID-19 were more likely to have mixed experiences compared to those with positive experiences. Our findings underscore the crucial role of financial security and social connections in reducing economic and emotional challenges older adults are facing during this crisis.

